# Giant Magnetoresistance: Basic Concepts, Microstructure, Magnetic Interactions and Applications

**DOI:** 10.3390/s16060904

**Published:** 2016-06-17

**Authors:** Inga Ennen, Daniel Kappe, Thomas Rempel, Claudia Glenske, Andreas Hütten

**Affiliations:** 1 Faculty of Physics, University of Bielefeld, P.O. Box 100131, 33501 Bielefeld, Germany; dkappe@physik.uni-bielefeld.de (D.K.); trempel@physik.uni-bielefeld.de (T.R.); huetten@physik.uni-bielefeld.de (A.H.); 2 Sensitec GmbH, Georg-Ohm-Straße 11, 35633 Lahnau, Germany; Claudia.Glenske@sensitec.com

**Keywords:** giant magnetoresistance, granular GMR, automotive applications, biosensors, nanoparticular sensors

## Abstract

The giant magnetoresistance (GMR) effect is a very basic phenomenon that occurs in magnetic materials ranging from nanoparticles over multilayered thin films to permanent magnets. In this contribution, we first focus on the links between effect characteristic and underlying microstructure. Thereafter, we discuss design criteria for GMR-sensor applications covering automotive, biosensors as well as nanoparticular sensors.

## 1. Introduction

It has been almost 30 years since one of the most fascinating advances in solid state physics occurred, the discovery of the giant magnetoresistance effect (GMR) by Grünberg and Fert in 1988 [1,2]. In thin metallic film systems, they observed that the magnetization of adjacent ferromagnetic films, separated by a thin non-magnetic interlayer, spontaneously align parallel or antiparallel, depending on the thickness of the interlayer. The orientation of the magnetization in the ferromagnetic layers strongly influences the resistance of the system. A parallel orientation is characterized by an electrical state of low resistance, while an antiparallel orientation is a state of high resistance. Due to the fact that the spacer layer thickness determines the initial configuration, an initially antiparallel orientation can be realized. The charm of this system lies in the fact that it enables a sensing of external magnetic field strengths in electrical units in between the two electric states of resistance. This discovery triggered an extensive research activity in this field in order to understand the underlying physical phenomenon as well as to exploit its technological potential. A remarkably short period, only a decade, lies between the discovery of the GMR effect and its first commercial realization in the form of magnetic field sensors and hard-disk read-heads [3]. Nowadays the spectrum of successful applications of GMR technology is impressively broad, ranging from applications in the air- and space or automotive industry, non-destructive material testing, or the compass functionality in mobile phones to biomedical techniques, like biometric measurements of eyes and biosensors, e.g., for the detection of viruses [3,4,5]. Thus, the potential of magnetoresistive technology seems to be far from being exhausted.

Nowadays the underlying physics of GMR and the interlayer exchange coupling are broadly understood. Nevertheless, when it comes to detail, discrepancies between experimental observations and theoretical models can arise: a realistic theoretical description of electron scattering at lattice discontinuities, disorder or defects is still a crucial factor [6,7].

In this review, we intend to provide an overview of different aspects of the GMR effect. The first section will focus on some of the ideas used to describe GMR effects theoretically in multilayers and to extend them into granular systems. Thereafter, we will have a look at different systems in which GMR can occur, with emphasis on the application-relevant side.

## 2. Theory

### 2.1. Giant Magnetoresistance in Magnetic Multilayered Systems

**Figure 1:**
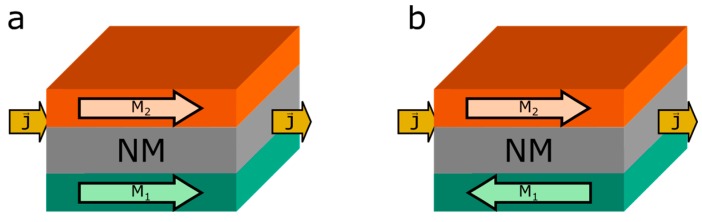
GMR double layer in Current in Plane (CIP) configuration. (**a**) Layer magnetization parallel; (**b**) antiparallel in respect to each other.

The giant magnetoresistance effect is the change of electric conductivity in a system of metallic layers when an external magnetic field changes the magnetization of the ferromagnetic layers relative to each other. A parallel alignment, like it is depicted in Figure 1a, has usually a lower resistance than an antiparallel alignment, Figure 1b. The effect size is defined as:

(1) $$\frac{\Delta R}{R}=\frac{R_{↑↓}-R_{↑↑}}{R_{↑↑}}$$

where $R_{↑↑}$ and $R_{↑↓}$ are the resistivity’s for parallel and antiparallel alignment, respectively. Alternatively the ratio is sometimes defined with $R_{↑↓}$ as denominator. The effect originates from spin-dependent transport of electrons in magnetic metals.

This section will introduce the Boltzmann equation approach for treating the GMR effect in multilayers in a classical picture. There are also a lot of publications presenting quantum mechanical treatments of the GMR, which will not be discussed here. The Kubo formalism [8] uses linear response theory to calculate the effect of small electric fields on currents. Examples for this ansatz are works by Camblong [9], Camblong, Levy and Zhang [10] and Levy, Zhang and Fert [11]. A detailed description and additional literature may be obtained in the extensive treatment of CPP GMR in multilayers by Gijs and Bauer [12].

**Figure 2:**
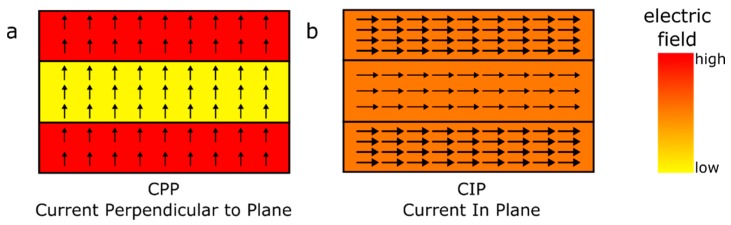
Simple double layer stacks in CPP (**a**) and CIP (**b**) configuration. CPP leads to a homogeneous current density (arrows) while the electric field is inhomogeneous, where CIP exhibits a homogenous electric field and an inhomogeneous current density.

The semi-classical Boltzmann equation is used to describe the transport of electrons in metals. The model builds on the work of Fuchs and Sondheimer who used it to calculate the dependence of film thickness on the conductivity of thin metal films [13,14]. The Boltzmann theory describes the distribution of carriers, here electrons, of wave vector *k* in vicinity of position **r** with the distribution function $f_{k}\left(r\right)$. The distribution function changes through processes of diffusion ${\left(\frac{\partial f_{k}\left(r\right)}{\partial t}\right)}_{diff}$, the influence of the external field ${\left(\frac{\partial f_{k}\left(r\right)}{\partial t}\right)}_{field}$ and due to scattering ${\left(\frac{\partial f_{k}\left(r\right)}{\partial t}\right)}_{scatt}$. The total rate of change vanishes in the steady state case which leads to:

(2) $${\left(\frac{\partial f_{k}\left(r\right)}{\partial t}\right)}_{diff}+{\left(\frac{\partial f_{k}\left(r\right)}{\partial t}\right)}_{field}=-{\left(\frac{\partial f_{k}\left(r\right)}{\partial t}\right)}_{scatt}$$

or after inserting the suitable expressions:

(3) $$v_{k}\cdot \nabla f_{k}\left(r\right)+e\left(\frac{\partial f_{k}\left(r\right)}{\partial E_{k}}\right)v_{k}\cdot E=-{\left(\frac{\partial f_{k}\left(r\right)}{\partial t}\right)}_{scatt}$$

with $v_{k}$ the velocity, $E_{k}$ the energy, e the charge of the electrons and E the electric field. At this point the description varies depending on the system at hand. In case of a Current In Plane (CIP) geometry, see Figure 2b, where the current is applied parallel to the layers, the electric field E will be homogenous throughout the layers, which simplifies the equation significantly. In case of a Current Perpendicular to Plane (CPP) geometry, see Figure 2a, the electric field differs from layer to layer. This description will be limited on the simpler CIP case, a treatment of the CPP geometry can be derived from [15].

Assuming that the electric field introduces just small perturbations into the electron distribution one can separate $f_{k}$ into:

(4) $$f_{k}\left(r\right)=f_{k}^{0}+g_{k}\left(r\right)$$

where $g_{k}\left(r\right)$ represents the deviation of the distribution from the equilibrium distribution $f_{k}^{0}$ which is given by the Fermi-Dirac distribution $f_{k}^{0}={\left[1+\exp\left(\frac{E_{k}-E_{F}}{kT}\right)\right]}^{-1}$. Furthermore, assuming negligible temperatures, spin-flip scattering can be omitted which governs the scattering term:

(5) $${\left(\frac{\partial f_{k}\left(r\right)}{\partial t}\right)}_{scatt}=\underset{k\prime }{\sum }\left[P_{kk^{\prime }}\left(1-f_{k}\right)f_{k^{\prime }}-P_{k^{\prime }k}\left(1-f_{k^{\prime }}\right)f_{k}\right]$$

with $f_{k}$ being shorthand for $f_{k}\left(r\right)$, $P_{kk\prime }$ being the probability of a electron of momentum k being scattered into k′ and vice versa. The principle of microscopic reversibility, meaning $P_{k\prime k}=P_{kk\prime }$, inserting Equation (4) and assuming elastic scattering only lead to:

(6) $${\left(\frac{\partial f_{k}\left(r\right)}{\partial t}\right)}_{scatt}=\underset{k\prime }{\sum }P_{kk^{\prime }}\left(g_{k^{\prime }}\left(r\right)-g_{k}\left(r\right)\right)$$

The scattering term may be simplified further by introducing the relaxation time $\tau_{k}=\underset{k\prime }{\sum }P_{kk\prime }$, which neglects the scattering-in processes. This relaxation time approximation decouples the Boltzmann equations and a linearization by discarding the second order term $Eg_{k}\left(r\right)$ leads to the linearized Boltzmann equation:

(7) $$v_{k}\cdot \nabla g_{k}\left(r\right)+eE\cdot v_{k}\left(\frac{\partial f_{k}^{0}\left(r\right)}{\partial E_{k}}\right)=-\frac{g_{k}\left(r\right)}{\tau_{k}}$$

Solving this equation for $g_{k}\left(r\right)$ leads to the electric current density J(r):

(8) $$J\left(r\right)=-\frac{e}{\Omega }\underset{k}{\sum }v_{k}g_{k}\left(r\right)$$

with Ω the systems volume. Assuming that $g_{k}\left(r\right)$ is a distribution depending on the x direction, the direction parallel to the current, and splitting $g_{k}\left(r\right)$ into a term with the velocity z component being positive $g_{k}^{+}\left(r\right)$ or negative $g_{k}^{-}\left(r\right)$ $g_{k}\left(r\right)=g_{k}^{+}\left(r\right)+g_{k}^{-}\left(r\right)$, the general solution of Equation (7) is:

(9) $$g_{k}^{\pm }\left(x\right)=e\tau_{k}\;E\cdot v_{k}\frac{\partial f_{k}^{0}\left(r\right)}{\partial E_{k}}\left[1+A_{k}^{\pm }\;\exp\left(\frac{\mp x}{\tau_{k}\lfloor v_{x}\rfloor }\right)\right]$$

The coefficients $A_{k}^{\pm }$ are given by the boundary conditions set at the outer surfaces and the interior interfaces. Derivations may also be found in [16].

An extensive treatment of this approach in the CIP geometry is given by Hood and Falicov [17]. They use specular and diffusive scattering at outer boundaries, tuned with the parameter $1\;>\;P_{\sigma }\;>\;0$ where 1 equals complete specular scattering. The metal interfaces allow for transmission parameter $T_{\sigma }$ and reflection $R_{\sigma }=1-T_{\sigma }$, which both might be specular or diffusive depending on the parameter $1\;>\;S_{\sigma }\;>\;0$. Furthermore they examined cases where relaxation times where identical $\tau_{k}$ for all layers and spins, the magnetic layers where equally thick $d_{F}$ and the electrons effective masses m. They found the following:

(a) $\frac{\Delta R}{R}$ increases with increasing specular scattering at the outer boundaries as long as the scattering at the interfaces is not completely specular for both spin channels.
(b) $\frac{\Delta R}{R}$ is in general small as long as the type of scattering at interfaces for both spin channels is equal $S_{\sigma =↑}=S_{\sigma =↓}$.
(c) $\frac{\Delta R}{R}$ dependence on the thickness $d_{F}$ of the magnetic layers is in general dependent on the scattering parameters, but its asymptotic value as function of $d_{s}$, the non-magnetic layer’s thickness is zero $\frac{\Delta R}{R}\left(d_{s}\to \infty \right)=0$, as well as for $\frac{\Delta R}{R}\left(d_{F}\to \infty \right)=0$.
(d) $\frac{\Delta R}{R}$ increases with increasing relaxation time τ to a maximum and then stays constant, or slowly decreases when the difference in specular scattering chances $S_{↑}$ and $S_{↓}$ are high.

For CPP geometry Valet and Fert found that spin-dependent scattering at the interfaces is the main contribution to GMR as long as the layers are thin, *i.e.*, for thicknesses of a couple of hundreds of angstroms, the contribution from bulk scattering becomes predominant [15]. In contrast to previous CIP treatments, the CPP geometry gives rise to an interface resistance. Furthermore the electrons of the minority spin accumulate at the magnetic interfaces and increase the spin-flip chance of electrons into the majority conduction band. Additionally this disparity is decreased by reversed spin-flip scattering, which is accounted to by introducing a spin diffusion length $l_{sf}$. For a spin-diffusion length $l_{sf}$ much higher than the layer thickness, a simple resistor scheme was found to be an adequate description of the process, which leads to a GMR effect of:

(10) $$\frac{\Delta R}{R}=\frac{R_{p}-R_{ap}}{R_{ap}}=\frac{{\left(R_{↑}-R_{↓}\right)}^{2}}{4R_{↑}R_{↓}}$$

with $R_{p}$ and $R_{ap}$ the resistances of the layered system with parallel and antiparallel magnetizations respectively and $R_{↑}$ and $R_{↓}$ the resistivity of the majority and minority electrons in a magnetic layer.

Lastly Ustinov and Kravtsov presented a unified theory of parallel and perpendicular GMR based on the Boltzmann equation [18]. They found CPP GMR to be higher than CIP GMR in most cases, but no definite relation between both. They found GMR even if the magnetic layers are not aligned antiparallel in zero magnetic field, in case the angle between magnetizations is exceeding a critical angle. The dependence of the GMR effect on the applied magnetic field was found to be different in CIP and CPP cases, while ${\left(\frac{\Delta R}{R}\right)}_{CIP}\left(H\right)={\left(\frac{R\left(0\right)-R\left(H\right)}{R\left(H\right)}\right)}_{CIP}<\mu^{2}$, with $\mu =\frac{M\left(H\right)}{M_{S}}$ being the relative magnetization, no such limit exists in the CPP geometry.

### 2.2. Giant Magnetoresistance in Granular Solids

**Figure 3:**
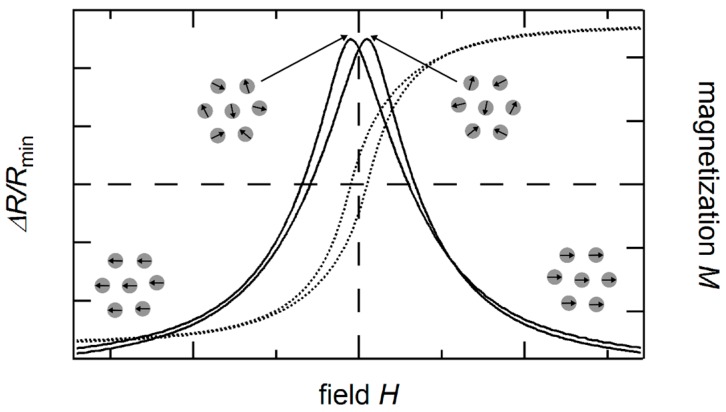
Schematic illustration of the granular GMR (solid line) in dependence of the applied field and sample magnetization (dotted line). The granular GMR exhibits the highest resistance at the coercive field as the magnetic moments of the particles are randomly oriented there. The dashed lines are a guide to the eye.

The giant magnetoresistance effect is not exclusively found in magnetic multilayers, but may also be found in systems with multiple ferromagnetic moments, which align parallel in exterior magnetic fields. An example of this are granular systems of a conducting non-magnetic matrix with embedded magnetic, conducting particles. In general, these systems have, without the influence of an external magnetic field, a random distribution of magnetic domains, caused by dipole interaction and depending on the distances between particles, Ruderman-Kittel-Kasuya-Yoshida (RKKY) coupling. By applying an external field, magnetic particles can be aligned in the corresponding direction, resulting in a decrease of resistance of the overall granular systems (see Figure 3). It was found in experiments, that the global relative magnetization $\mu \left(H\right)=\frac{M\left(H\right)}{M_{S}}$ is a good variable to describe the GMR in granular systems:

(11) $$\frac{R0\left(H\right)-R\left(H\right)}{R\left(H\right)}\approx A\;\mu^{2}\left(H\right)$$

where A determines the effect amplitude and is to be measured for each experimental setup separately [19].

A couple of models exist, which try to evaluate the parameter A on a theoretical basis. Kim *et al.* [20] proposed a model based on the Kubo formalism. They modeled the magnetic grains as centers for potential barriers. They found their model to be in agreement with data by Xiao, Jiang and Chien [19], but as μ approaches 1, the GMR deviated from $\frac{\Delta R}{R}\propto \mu^{2}\left(M\left(H\right)\to M_{S}\right)$. Additionally, they examined the GMR dependence on grain size compared to experiments by Xiao *et al.* [21] and Xiong *et al.* [22]. They found an optimal size for grains (compare Figure 14). The GMR effect rises rapidly until it reaches a maximum at the optimal grain size and then slowly decreases. They assumed this to be an effect of larger grains acting as conduction medium instead of only scattering centers.

Zang and Levy using a CPP like formalism they derived previously [23,24]. They found:

(a) Magnetoresistance increases with the mean free path of the electrons in the matrix material.
(b) Magnetoresistance increases with the ratio between spin-dependent and spin-independent potentials, which they expect to be comparable to those found in multilayers.
(c) Magnetoresistance increases with spin-dependent scattering roughness of the interfaces.
(d) Magnetoresistance increases with decreasing grain size as long as the external magnetic field is strong enough to saturate all granules.
(e) Magnetoresistance increases with concentration of granules as long as the granules do not form magnetic domains at high concentrations.
(f) Magnetoresistance depends on the size distribution of the grains and needs to be precisely known to compare theory and experiment.
(g) Magnetoresistance differs from $\frac{\Delta R}{R}\approx A\;\mu^{2}\left(H\right)$ when the grain size distribution is broad as μ approaches 1.

Ferrari, da Silva and Knobel found that granular systems exhibits a behavior similar to the CPP GMR in multilayers for the case of the granule conductivity being much larger than the conductivity of the matrix [25,26].

**Figure 4:**
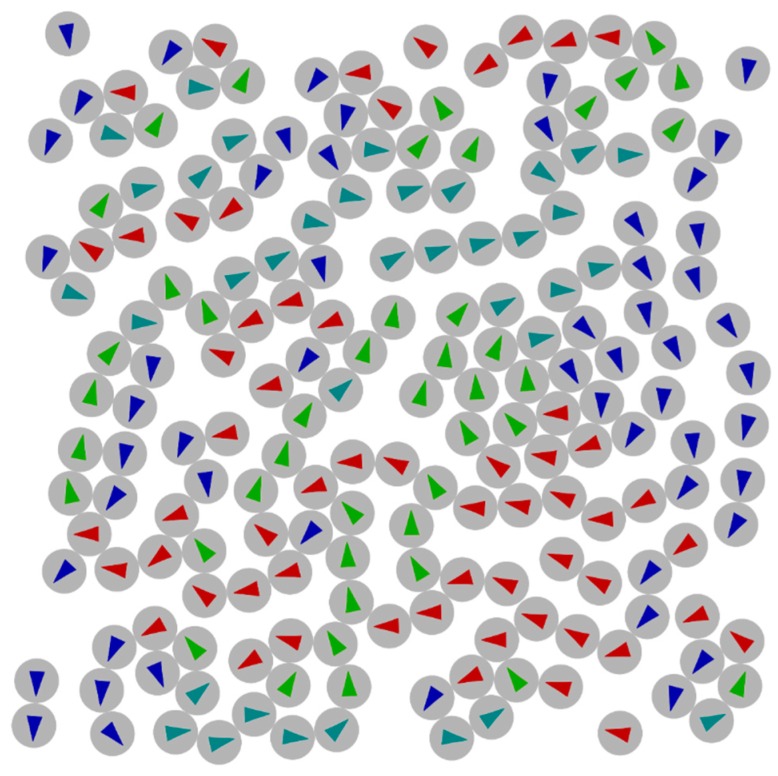
Micromagnetic simulation of nanoparticles (20 nm) combined with a molecular dynamics simulation to model clustering of particles, see [27].

These models all use some kind of averaging the magnetic moments of the systems, which seems to work fine as long as the concentration of grains is low enough. As soon as the distance between grains becomes small their dipole interactions lead to the assembly of ferromagnetic or antiferromagnetic domains, or more complex ordering. Teich *et al.* [27] used micromagnetic simulations to calculate magnetic ground states for magnetic particle assemblies, an example may be seen in Figure 4. These areas of magnetic ordering are likely to have influence on the electric conductivity of the system. To the best of our knowledge, there are to this point no studies on the influence of this. Systematic addition of differently shaped particles or the removal of particles could lead to increased GMR and tailoring of a granular system to specific needs.

## 3. GMR Systems

### 3.1. Thin Film Systems

**Figure 5:**
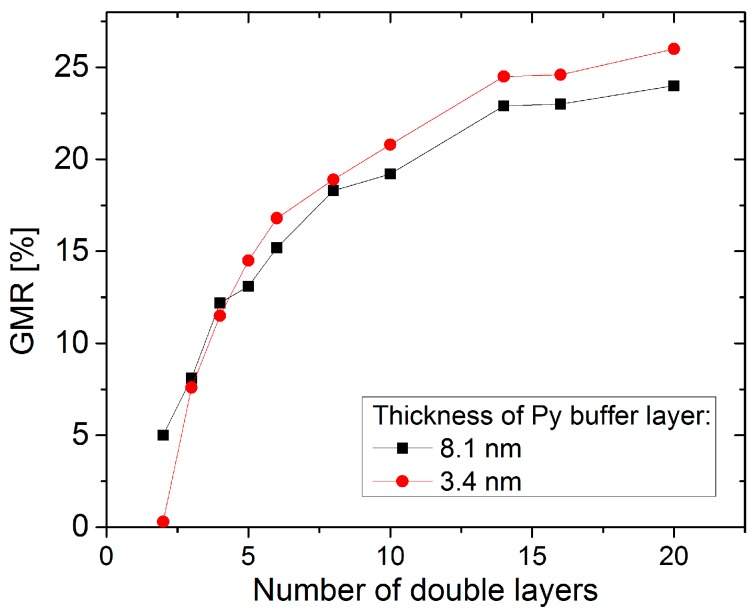
GMR amplitude measured at room temperature as a function of the number of double layers N of (Co_1.1 nm_/Cu_2.0 nm_)_N_ for two Py buffer layer thicknesses of 3.4 nm (red) and 8.1 nm (black), respectively. Data taken from [33].

The first GMR multilayer stack was prepared in 1988 by Fert *et al.* [1]. They examined the characteristics of a {Fe/Cr}_N_ system to explore the origin of the GMR effect. Driven by possible applications as sensors in automotive and read-head industry, numerous studies have been performed to improve the GMR characteristic since then [6,7,28]. A main goal was the improvement of layer materials and thicknesses in order to identify the optimum microstructural and magnetic features which enhances the GMR effect amplitudes in the multilayer systems and therefore, achieve higher sensitivities for sensor applications. Interface roughness is one of these microstructural characteristics that determines the GMR potential and has been intensively studied (for a review of numerous interface studies performed on Fe/Cr and Co/Cu multilayers see [6]). Furthermore the grain size has to be considered [29,30]. It has been found that neither the crystallite size nor the interface roughness alone determine the GMR of a multilayer, but the combination of both aspects. A combination of large grains with moderate interface roughness has been reported to be an ideal candidate for good GMR [29,31,32]. The interface roughness can be influenced employing a suitable buffer layer, whereas an appropriate buffer layer thickness has to be chosen depending on the materials used and the number of double layers. In Figure 5 the influence of the number of Co_1.1nm_/Cu_2.0nm_ double layers on the GMR amplitude has been shown for two different Py buffer layer thicknesses. For small numbers of bilayers an increasing thickness of the buffer layer is favorable to obtain a larger GMR amplitude due to the enhancement of the antiferromagnetic coupling in the undermost bilayers.

This concept fails when sputtering a large number of bilayers, because the shunting of the thicker buffer or bilayer compensates or even destroys the effect of a larger antiferromagnetically coupled layer fraction [33]. However, due to the high GMR magnitude and, therefore, sensitivity for small changes of magnetic fields, GMR systems are very attractive for sensor applications in industry. In the following section we will have a closer view on different GMR applications:

#### 3.1.1. Information Technology

**Figure 6:**
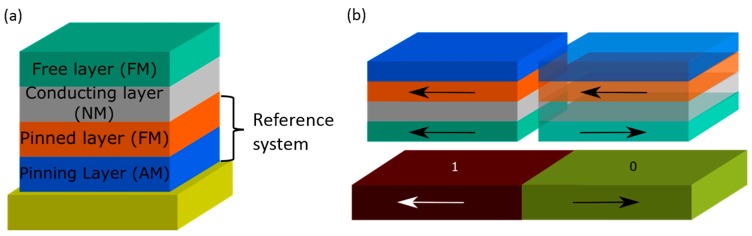
(**a**) Schematic setup of the stack configuration of a GMR spin-valve sensor; (**b**) Conceptual operation of a GMR read head: when a spin-valve sensor moves across an interfaces between two bits with magnetic moments oriented in opposite direction (marked by “1” and “0”), the magnetic moment of the free layer is reoriented according to the orientation of the next bit.

The first industrial application of GMR thin film systems after the discovery of the effect was in the field of information technology: the realization of GMR based hard-disk read-heads in 1997 [3,28]. Here, the GMR sensor is used to detect the magnetization direction of the bits on the magnetic recording medium, which are assigned to a logical 0 or 1, respectively. Due to the continual improvement of storage density, and thus reduction of bit size, a good scalability and high sensitivity of the sensor element are necessary requirements. Furthermore, a linear sensor characteristic for the reliable detection of bits and long-term stability are crucial factors. To detect the transition between bits GMR spin-valve sensors are commonly used, which have been first proposed by Dieny *et al.* [34]. As schematically shown in Figure 6a, these spin-valves consist of three functional layers: a ferromagnetic (FM) layer with a fixed direction of magnetization (reference system), a non-magnetic (NM) interlayer and another ferromagnetic layer, which magnetization direction can freely align with external magnetic fields (free layer). To achieve a maximum stability of the reference system against external fields, it typically consists of an artificial antiferromagnet (AM) with a pinned layer and an antiferromagnetically coupled reference layer. That way, the magnetization of the reference layer can be fixed into a certain direction, employing the exchange bias effect [35]. The exchange bias field is temperature dependent and varies for different materials. In order to let the free layer follow changes in the external magnetic field, the thickness of the non-magnetic interlayer has to be chosen to ensure a minimal magnetic coupling of the magnetic layers.

Moving a spin-valve across the interface between two bits with opposite magnetization direction, the orientation of the magnetization of the free layer changes according to the stray field of the bits, resulting in a resistance change of the entire reading structure (compare Figure 6b). The resistance change causes a variation of current flowing through electronic circuits connected to the reading structures. This change of the current is detected and decoded to reveal the information stored on the disk.

**Figure 7:**
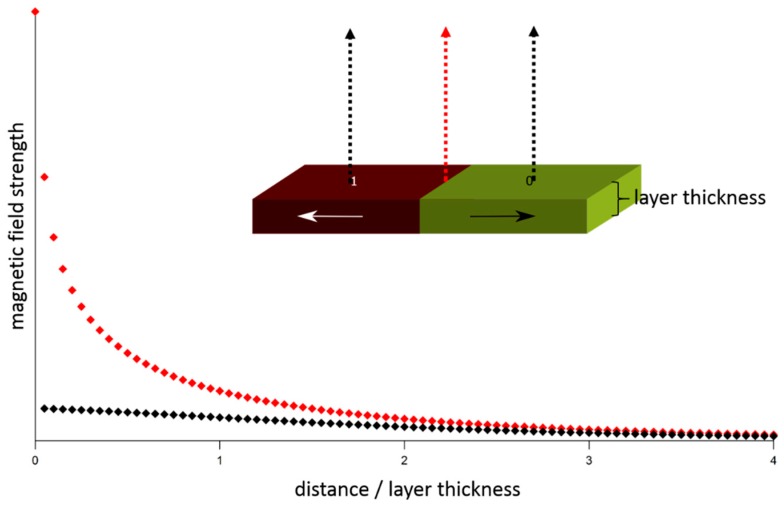
Magnetic stray field strength as a function of the distance from the layer surface, calculated for a bit structure with opposing magnetic moments as shown in the sketch. The arrows in the sketch mark the positions of the stray field calculations (black curve: middle of bits, red curve: interface between bits).

For sensing small magnetic fields the distance between the stray field source and the sensor element is an important parameter, because the stray field strength drops strongly with increasing distance [36]. In Figure 7 the magnetic stray field strength as a function of the distance *z* is shown, illustrating the 1/*z*^3^ dependence. Therefore the reading head is required to maintain a constant distance to the spinning hard disk surface, which has to be as small as possible.

Recently, hard disk drives came onto market, which use He as filling gas between disks and read heads to reduce turbulences, and thus allowed a reduction of the distance between the disks and their read heads. Combined with e.g., the shingled magnetic recording technique for hard disks, where data tracks overlap with the adjacent tracks like shingles, GMR technology allows one to realize hard disk drives with storage capacities of up to 10 TByte [37].

#### 3.1.2. Automotive Applications

The automotive industry offers a great field of applications for GMR sensors like sensing rotational speed, angle and position [38,39,40]. Several technical requirements have to be fulfilled to make the GMR technology compatible for automotive applications: linear and non-hysteretic GMR characteristics, high sensitivity, small temperature drift and long-term stability under application conditions. For application in rotational speed sensing for example, spin-valve sensors are commonly used (see Section 3.1.1) to ensure the desired sensor characteristics and sensitivity for small magnetic fields. For this purpose, the free layer of the spin-valve system needs to have an anisotropy axis, to which the magnetization preferably orients, if no external magnetic field is applied. This axis can be realized by using crystal anisotropy or by adjusting the geometry of the GMR structure and making use of the shape anisotropy. To obtain a high anisotropy and therefore a strong alignment, a high aspect ratio of the GMR structure has to be achieved. For example, for realization of linear transition regions in the range of several mT, the width of the GMR device has to be structured down to sizes of 1 µm and below [41,42]. A configuration which considers these aspects is the arrangement of meander shaped GMR sensors in a Wheatstone bridge [43]. This configuration minimizes the effects of temperature and disturbing magnetic fields. Furthermore, in this configuration hysteresis effects can be minimized e.g., by a slight change of the pinning directions out of the primary 90° orientation. In [43] a reduction of hysteresis by about 1/5 of the primary value has been reported. However, due to this geometry the GMR sensitivity is decreased and finally, for the optimization of GMR sensors always a compromise between sensitivity and magnetic reversal characteristic have to be found in consideration of the application of the sensor.

Since a lot of automotive magnetic sensors are implemented into security-relevant functions, it is of importance that the magnetic behavior of the GMR sensors be stable under the applied conditions. Thermal stability is a main factor here due to the exposure to high temperatures in the range of 200–360 °C during manufacturing as well as temperatures up to 200 °C for extended periods during up to 40,000 h of operation, which have to be tolerated by the sensor without loss of performance. Many studies report an initial increase of the GMR magnitude, compared to the as prepared samples, after an annealing for a short time at moderate temperatures between 250 °C and 380 °C [44,45,46,47,48,49]. This increase of the GMR effect originates from an improvement of the quality of the interfaces between the magnetic/non-magnetic layers as well as defect recovery by diffusion processes [45,48,50].

**Figure 8:**
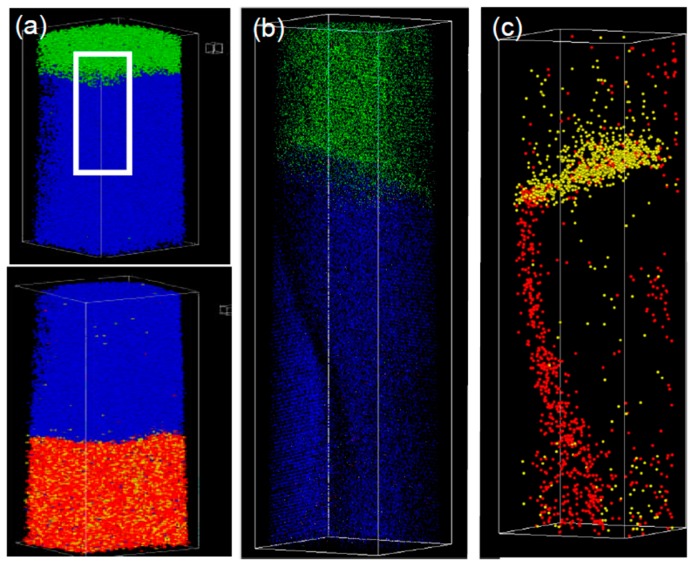
3D reconstruction of atom probe tomography of a Fe (red) Ni (yellow)/Cu (blue)/Co (green) trilayer: (**a**) as prepared Co/Cu interface (upper image) as well as Cu/Py interface (lower image); (**b**,**c**) show the element distribution after annealing at 350 °C for 30 min for the marked Co/Cu interface region in (**a**) (adapted from [51]).

The optimum temperature depends on the choice of layer materials, thicknesses, the possibly used buffer layer and substrate materials. Within the framework of this review the focus is on Co/Cu based layer systems. For example, if the thickness of the individual layers has been optimized for the first antiferromagnetic coupling (AFC) maximum an optimum temperature of about 150 °C has been reported [52], while for systems optimized for the second AFC maximum a critical temperature of about 375 °C has been observed [53]. For annealing processes above the critical temperature a breakdown of the GMR amplitude is observed. Different reasons for this deterioration of GMR in Co/Cu multilayers have been discussed in literature: Observations of Co bridges through Cu layers have been reported by means of field ion microscopy and transmission electron microscopy (TEM) [54,55]. These defects of the layered structure were observed in systems with high interface roughnesses even in the as prepared state leading to a strong ferromagnetic coupling of the adjacent Co layers. TEM studies of Co/Cu multilayer samples reported by Rätzke *et al.* show the transport of Cu into the Co layers along grain boundaries [47]. An alternative method for the observation of the mechanism of GMR deterioration is the atom probe tomography (APT) [51,56,57]. In Figure 8a a 3D reconstruction of a Py_25nm_/Cu_20nm_/Co_10nm_ trilayer obtained by APT is shown. After an annealing at 350 °C for 30 min. (Figure 8b,c) it can be clearly seen that Ni atoms from the Py buffer layer segregate along grain boundaries into the Cu layer (red dots in Figure 8c). This segregation path forms the initial stage of pinhole formation and causes ferromagnetic bridges through the non-magnetic coupling layer, causing a decrease of GMR effect [51].

**Figure 9:**
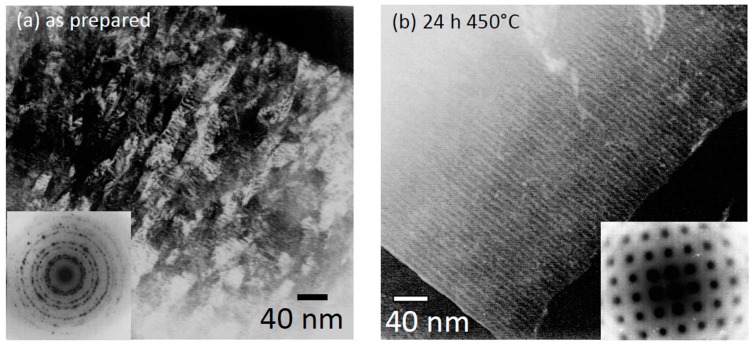

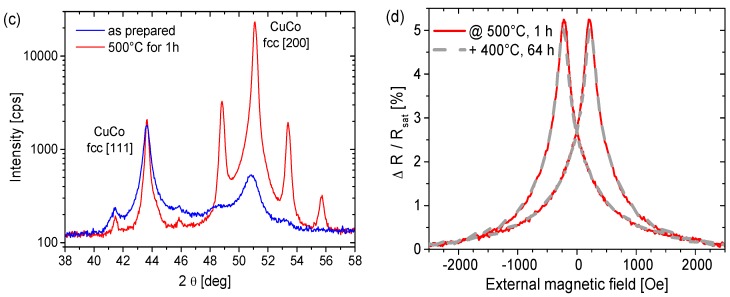
Comparison of TEM images of a [Py_3nm_/Cu_6nm_/Co_3nm_/Cu_6nm_]_20_ multilayer in the as prepared state (**a**) and after annealing at 450 °C for 24 h (**b**). The insets show the corresponding selected area diffraction pattern. The micrographs prove that the layered structure of the sample is preserved during annealing while the microstructure changes from polycrystalline to quasi single crystalline, oriented in fcc [100] direction; (**c**) X-ray diffraction pattern of a Co/Cu multilayer system before and after annealing showing the recrystallization effect; (**d**) GMR measurements at room temperature for the recrystallized Co/Cu multilayer: the GMR effect remains stable at further heat treatment at 400 °C for 64 h [33].

A concept how to avoid these effects and to improve the temperature stability of Cu/Co multilayer systems has been reported by Heitmann *et al.* [58]: For a [Py_3nm_/Cu_6nm_/Co_3nm_/Cu_6nm_]_20_ multilayer system it has been shown that an annealing at 500 °C for 24 h triggered a complete recrystallization of the sample from a dominating polycrystalline [111] texture in the as prepared state to a [100] quasi single crystalline state after annealing. The most striking aspect of the microstructural evolution is the preservation of the layered structure (compare Figure 9a,b). This crystallographic reorientation is triggered by the minimization of lattice mismatch elastic energy: Under equal strain the elastic energy in a [111] oriented CoCu material is higher than the energy in a [100] structure due to the elastic properties of the materials. By recrystallization in a [100] structure a reduction of elastic energy in the order of 0.8 eV per interface atom is achieved [33,59]. But it is important to note, that a prior annealing of the sample at moderate temperatures which has led to a considerable reduction of dislocations in the course of recovery, while the temperature was not high enough to activate recrystallization process, a further temperature increase not necessarily initiate a recrystallization any more. This is caused by the decrease of the driving force [60]. Therefore, recrystallization can only occur after heating up the sample directly to sufficient temperatures. The GMR measurements, given in Figure 9d, for the recrystallized Co/Cu multilayer show that the GMR effect remains stable at further heat treatment below the initial annealing temperature for 64 h.

#### 3.1.3. Biosensors

Due to the ability of GMR systems to sense even small magnetic fields, the potential of GMR sensors for the detection of magnetic beads was realized and led to another growing technological field, the development of magnetic biosensors for life science applications. Only ten years after the discovery of GMR the first magnetic biosensor was presented by Baselt *et al.* [61].

**Figure 10:**
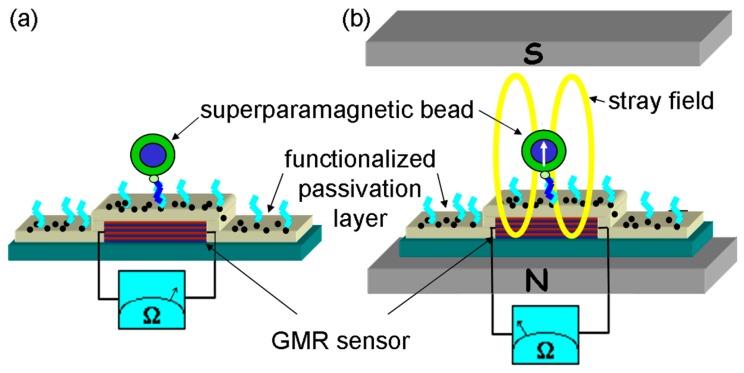
Schematic representation of a magnetic biosensor: (**a**) a superparamagnetic bead functionalized with a receptor molecule hybridize to the target molecule attached onto the sensor surface; (**b**) An external field align the magnetic moment of the bead and the magnetic stray field can be detected by the GMR sensor (adapted from [62]).

In Figure 10 an illustration of the detection principle is shown. Specific proteins are immobilized on the sensor surface. Superparamagnetic nanoparticles or beads, which are specifically attached to a target antibody, are used for detection. In a washing step, unbound magnetic markers are removed and beads bound to antigen molecules are measured. 

**Figure 11:**
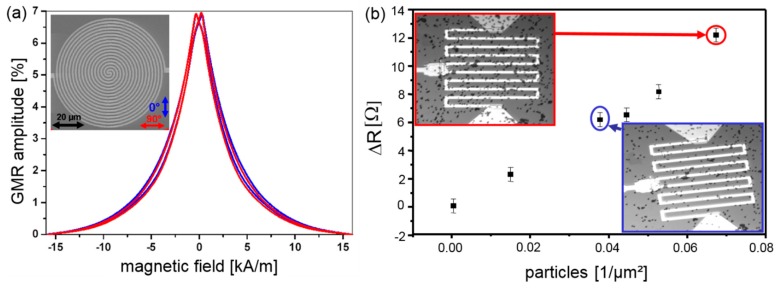
(**a**) Isotropic GMR characteristic measured at room temperature for a spiral shaped GMR sensor for two in-plane fields oriented perpendicular to each other; (**b**) Change in the resistance of meander shaped GMR sensors, each with an area of 100 × 100 µm^2^, as a function of particle density. The SEM images show the particle coverage of the sensors corresponding to the measurements marked by colored circles (data taken from from [62]).

The superparamagnetic nature of the beads allows to switch on their magnetic stray field by a homogeneous external magnetic field oriented perpendicular to the sensor surface, see Figure 10b. Hence, the stray field components of the magnetic markers within the sensitive sensor area can be detected by a drop in the electrical resistance of the GMR sensor. For an optimum bead detection, GMR sensors with isotropic signals and high sensitivities are needed. In [62,63] the use of a Py_1.6nm_ [Cu_1.9nm_/Py_1.6nm_]_10_/Ta_3nm_ multilayer stack for the detection of magnetic beads was reported. To prevent any influences of magnetic anisotropies of the used materials on the GMR characteristic a spiral-shaped structure has been chosen. In Figure 11a the nearly isotropic GMR characteristic for two perpendicular oriented in-plane magnetic fields are shown. For this type of sensor a sensitivity of 0.6% per kA/m for in plane magnetic fields has been achieved, resulting in a detection limit of a DNA concentration of only 16 pg/µL, which is superior to standard fluorescence detection methods [63]. The dependence of the resistance change ΔR on the particle coverage of the sensor surface is shown in Figure 11b. A nearly linear behavior of the output signal is observed for low particle concentrations [62].

On the way from a simple bead detection to a fully integrated, easy to use, hand held “lab on a chip” device for applications in human or veterinary diagnostics, several challenges have to be mastered: (1) The magnetic core of the magnetic markers has to be stabilized to preserve their magnetic properties. Usually, this is achieved by embedding superparamagnetic magnetite nanoparticles in a polymer matrix. Chemically synthesized FeCo nanoparticles are good candidates even for single molecule detection as well, due to their superior saturation magnetization and, therefore, larger stray fields [64]; (2) the interface between chemistry and biology has to be fitted for each application, to allow a specific functionalization of the marker and sensor surface, e.g., for the detection of biotin-labeled DNA, streptavidin coated particles can be used [65,66]; (3) the GMR sensors have to be incorporated in fluidic environments, which enable the magnetic markers to pass the sensor surfaces at close distances to ensure a binding onto the surface within an acceptable time scale [67]. Due to the magnetic nature of the markers, magnetic attraction forces, created e.g., by on-chip conducting lines or magnetically structured thin films, can be employed to pull beads towards the sensors [68,69,70,71,72,73,74,75]. Another way to concentrate beads on a sensing surface uses of ultrasonic standing waves inside a microfluidic channel system [76,77] or the microfluidic system itself can be utilized to transport beads towards the sensor surface, e.g., by designing a ramp like structure [67,78].

A new concept to transport the magnetic particles in a “lab on a chip” environment without the need of external forces like microfluidic pumps, is a magnetic on-off ratchet [79,80]. Here, a combination of asymmetric magnetic potential and Brownian motion of magnetic beads moves particles through the device. The asymmetric magnetic potential is achieved by combining an external magnetic field with a spatially periodic array of conducting lines. When the asymmetric field is applied, particles move towards the minima of the potential. After switching off the diffusion process starts. Due to the asymmetric shape of the potential the particles are transported to the next minima when the field is reactivated and thus, a net transport process is achieved [79].

**Figure 12:**
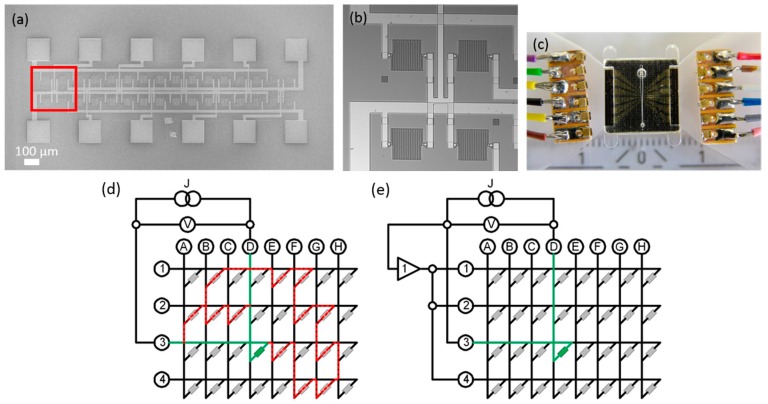
Lab-prototype of a “lab on a chip” device: (**a**) SEM image of the device heart consisting of 32 GMR sensors. The marked region of four meander shaped GMR sensors is shown enlarged in (**b**); (**c**) Photograph of the connected device; (**d**,**e**) illustrate the advantage of the guarding procedure for an analysis of a matrix of 32 sensors. Only the green labeled sensor element (line 3, column D) should be measured. Possible other paths of the current are marked in red; (**d**) the matrix without guarding; (**e**) when the guarding approach is applied.

The realization of a lab-prototype of a “lab on a chip” device is shown in Figure 12. An array of 32 meander shaped GMR sensors combined with a suitable microfluidic design, which optimizes the bead capture rate. The measurement of individual sensor coverage can be improved by application of the guarding procedure. This procedure employs an additional amplifier which switches the voltage on the adjacent sensor rows, enabling an equal potential of the rows (see Figure 12d,e). Provided that the resistances of the matrix elements are of the same magnitude and much larger than the resistance of the supply lines, the measurement current will not expand on other paths and every resistance in the sensor matrix can be addressed individually.

### 3.2. Granular Bulk Systems

**Figure 13:**
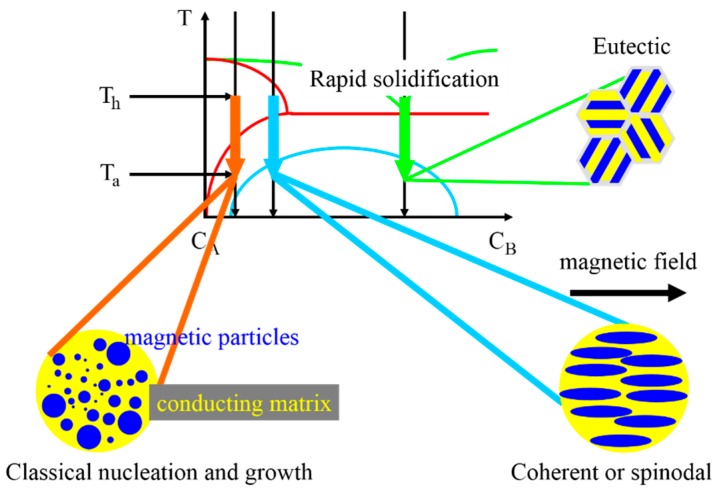
Schematic phase diagram of two metals A (magnetic) and B (non-magnetic) illustrating different types of decomposition which can lead to GMR effects in heterogeneous bulk alloys: (1) classical nucleation and growth of precipitates; (2) coherent or spinodal and (3) eutectic decomposition, which forms a lamellar microstructure similar to multilayers.

Four years after the discovery of the GMR effect in multilayer structures it was shown by Berkowitz *et al.* and Xiao *et al.* that GMR is not restricted to thin film systems, but occurs in heterogeneous bulk alloys, too [19,81]. Both groups utilized magnetron sputtering or melt spinning to create ferromagnetic Co precipitates in a non-magnetic Cu matrix, respectively. Underlying physical mechanisms which can induce the formation of such granular bulk GMR structures in alloys are summarized in the schematic phase diagram shown in Figure 13. Different decomposition types for the formation of magnetic precipitates (metal A) in non-magnetic matrix materials (metal B) have been observed: (1) decomposition by classical nucleation and growth, e.g., in Ag-Co systems [21,82,83]; (2) coherent or spinodal decomposition, e.g., in Cu-Co systems [84,85] and (3) eutectic decomposition, e.g., in Au-Co alloys [86,87]. However, it is expected that Ag-Co and Cu-Co systems will behave as decomposed due to a large content of Co [88].

By applying an external magnetic field during the decomposition process (see Figure 13, case 2), elongated magnetic precipitates can be prepared. This has been demonstrated by Hütten *et al.* in AlNiCo_5_ bulk alloys [89]. It has been shown that the probability of spin scattering is two times higher, if the direction of the current is perpendicular to the direction of the particle elongation.

**Figure 14:**
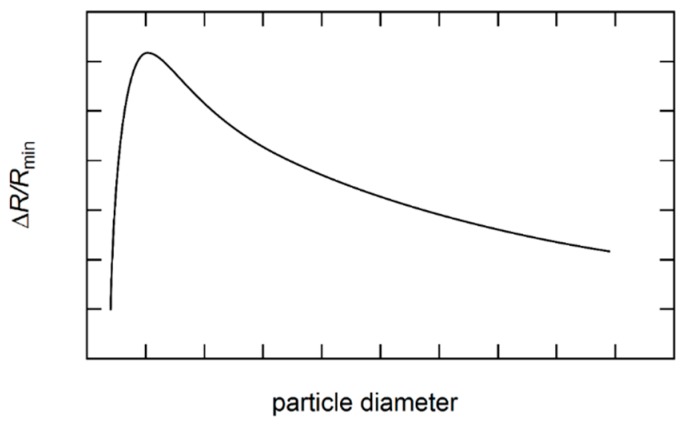
Schematic illustration of the granular GMR effect in dependence of the particle size.

The GMR characteristics in these granular systems is closely correlated to the magnetic behavior of the samples, as discussed in Section 2.2. Due to TEM investigations it is known that an annealing of granular systems causes a coarsening of the magnetic precipitates and an increase of interparticle distances as it has been reported for Cu-Co [19,88] and melt-spun Au-Co [87,90]. Furthermore, in [88] it has been confirmed by Lorentz microscopy that single domain Co particles exist in as-quenched Au_71.6_ Co_28.4_ ribbons, and multidomain Co particles in annealed ribbons, respectively. The changes in grain size and the formation of multidomain particles reflect in the magnetic measurements and, therefore, in the GMR characteristics. While a constant decrease of the granular GMR with increasing particle size was observed by some groups [81,91,92], it was found in refs. [19,93,94,95], that the granular GMR first increases up to a maximum value at about the electron mean free path λ and then decreases (see Figure 14). In both cases, the granular GMR decreases approximately with the inverse of particle size. It was concluded that the decrease of the granular GMR arises from the decreasing spin-dependent interfacial electron scattering as the surface to volume ratio decreases with increasing size [23,96]. Ge *et al.* concluded, that for low annealing temperatures, defects, disorder and mismatch stress are reduced [94]. Thus, the overall film resistance reduces, which leads to an increased granular GMR. At higher temperatures, particles grow fast enough compared to the curing of the film defects and the granular GMR degrades. Wang *et al.* noted on particle size dependence of the granular GMR, that it depends on whether the particles are superparamagnetic, single domain ferromagnetic or multidomain ferromagnetic. While their calculations show a constant decrease for superparamagnetic particles, it exhibits a maximum for single domain ferromagnetic particles [93].

The dependence of the granular GMR on the ferromagnetic volume fraction is comparable to the particle size dependency: At low ferromagnetic volume fractions, the particles are small and few in number, therefore only a small granular GMR is measurable. With increasing ferromagnetic volume fraction, the granular GMR increases until it reaches the optimum value at a ferromagnetic volume fraction between 15% and 30%, depending on the used material system. Thereafter, it decreases with an increasing ferromagnetic volume fraction as the particles become larger and more densely packed reducing the surface to volume ratio. Furthermore, multidomain particles can arise and dipole interactions between neighboring ferromagnetic particles become more important. Finally, particles form a large connecting network with ferromagnetic domains at the percolation threshold of 55% and only an anisotropic magnetoresistance (AMR) is observable [93,94,95,97,98,99,100,101,102,103,104].

Summarizing the findings of many studies, it can be stated that the size, distribution and amount of ferromagnetic particles as well as the interface roughness determines the resulting GMR effect in granular alloys [87,88,90,105]. Therefore, it is essential to control these parameters to improve the GMR effect in granular systems.

### 3.3. Hybrid Structures

**Figure 15:**
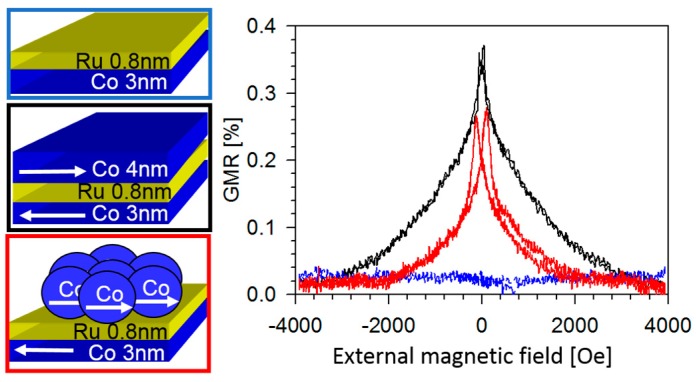
Proof of concept of the idea that Co nanoparticles can be coupled to a Co layer via a Ru spacer layer coupling. As references, the GMR characteristics of a Co_3nm_/Ru_0.8nm_/Co_4nm_ layered sample (black curve) and a Co_3nm_/Ru_0.8nm_ system (blue curve) are given. The resulting GMR curve (red) measured at room temperature of the Co_3nm_/Ru_0.8nm_/Co particles (diameter: 12 nm) hybrid system clearly shows a spin-valve character (adapted from [110]).

GMR is not restricted to thin films or bulk systems only. It also occurs in pure particular systems (see next section) and hybrid materials containing thin films as well as magnetic clusters. These hybrid structures can be prepared e.g., by heating of films or by preparing multilayers with ultrathin and therefore discontinuous magnetic layers [106,107,108,109,110]. Holody *et al.* showed that Co/Py hybrid systems reveal advantages for sensor applications like a lateral decoupling in the cluster layer in combination with a low coercive field [106]. Unfortunately, the above mentioned techniques for the preparation of hybrid materials typically lead to large cluster size distributions, making the investigation of influences like cluster size, distances and concentration on the resulting GMR characteristic hard to uncover. In [110] the idea to employ a “bottom-up” method by replacing a ferromagnetic electrode of a thin film trilayer by predefined magnetic nanoparticles has been presented. Here, a Co_3nm_/Ru_0.8nm_/Co_4nm_ thin film system has been prepared by sputtering as a reference, which shows a GMR amplitude of 0.36% at room temperature (see black curve in Figure 15). The thickness of Ru interlayer has been chosen according to the best interlayer exchange coupling. For preparation of the hybrid system, Co nanoparticles with a mean diameter of 12 nm have been prepared via a wet chemical synthesis [111,112]. A monolayer of these particles have been spin coated on top of the Ru interlayer, thus replacing the 4 nm thick Co film as magnetic electrode. The corresponding GMR characteristic (see red curve in Figure 15) shows a similar behavior compared to the reference system with an effect amplitude of 0.28% at room temperature. 

This indicates that the magnetic Co nanoparticles can be coupled to a Co layer utilizing the spacer layer coupling. The smaller saturation field of the hybrid structure indicates a smaller spacer layer coupling compared to the layered system of the same spacer layer thickness, but due to the larger magnetic moment of the 12 nm sized Co nanoparticles compared to the 4 nm thick Co film, the contribution of the Zeeman energy is higher, too. Thus, in the case of the hybrid system is the saturation field is smaller than for the layered structure for an assumed equal coupling strength. Nevertheless, this method seems to allow a finer tuning of GMR characteristics of hybrid systems, which is of great interest from an application point of view.

### 3.4. Nanoparticular GMR Systems

**Figure 16:**
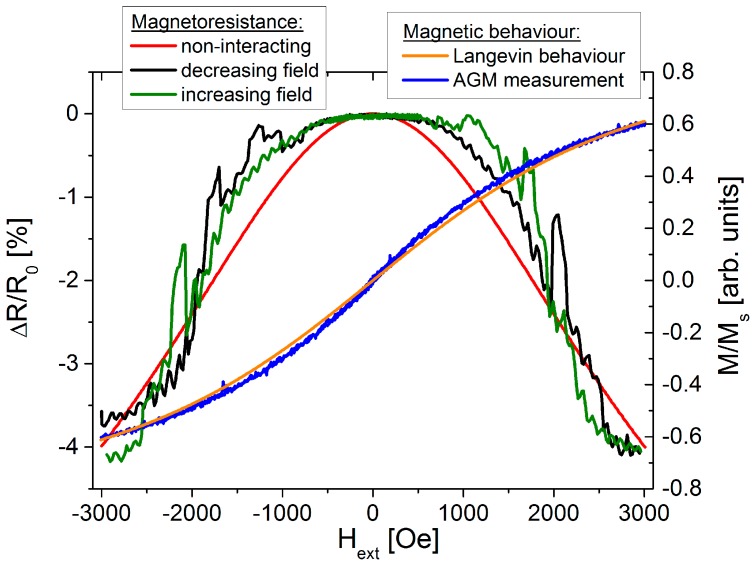
GMR characteristic of a monolayer of 8 nm sized Co nanoparticles measured at room temperature with an in-plane external magnetic field (sample current: 1 mA, R_0_: 1.6 kΩ). The measurement is compared to the expected behavior of non-interacting particles (red curve). Additionally, the corresponding magnetic measurement is shown (blue curve) (adapted from [121]).

Typically, granular materials are prepared by top-down methods such as co-sputtering or co-evaporation of matrix and precipitated materials as well as by metallurgic techniques [97,113,114,115]. Particle size, volume fraction and magnetic configuration of the particles have to be controlled due to the GMR’s dependence on these parameters. These requirements can be fulfilled more easily by employing bottom-up approaches for the preparation of the granular systems like the embedment of prefabricated magnetic nanoparticles into non-magnetic matrix materials. This approach has been applied at first by Dupuis *et al.*, who used in the gas-phase prefabricated Co and Fe particles simultaneously deposited with Ag as matrix material onto cold substrates [116]. This technique allows the independent variation of particle size and volume ratios and therefore the study of the dependence of GMR on these parameters. Furthermore, different material systems can be realized in a simple manner [116,117,118]. In 2007 Tan *et al.* showed that chemically synthesized ligand stabilized magnetic FeCo nanoparticles can be used for a preparation of magnetoresistive granular super-crystals [119]. In this case, the electrically isolating ligand shell acts as a tunneling barrier. Tunnel magnetoresistance effect amplitudes of up to 3000% at low temperatures have been reported for these nanoparticular systems [120]. In [121] such ligand stabilized nanoparticles have been used to create two-dimensional granular GMR structures. Therefore superparamagnetic Co nanoparticles with a mean diameter of 8 nm have been arranged in a monolayer onto a SiO substrate via a self-assembly process. The insulating ligand shells have been removed by an annealing process in a reducing gas atmosphere. Afterwards, without breaking the vacuum, a thin Cu layer has been deposited on top of the nanoparticles in order to establish an electrical contact between the particles. In Figure 16 a result of a GMR measurement at room temperature is shown. The bell shaped GMR characteristic follows mostly the expected behavior for non-interacting particles deduced from the magnetization reversal by Equation (11) (red curve, Figure 16). 

Aside from the expected magnetoresistance characteristic, additional features appear symmetrically for in- and decreasing external fields, which can be attributed to the inner magnetic arrangement in the particle assembly. Caused by a dipolar coupling of adjacent particles, magnetic domains can be formed with an antiparallel arrangement of magnetic moments which maintains a higher stability against external influences compared to the non-interacting particles [121].

**Figure 17:**
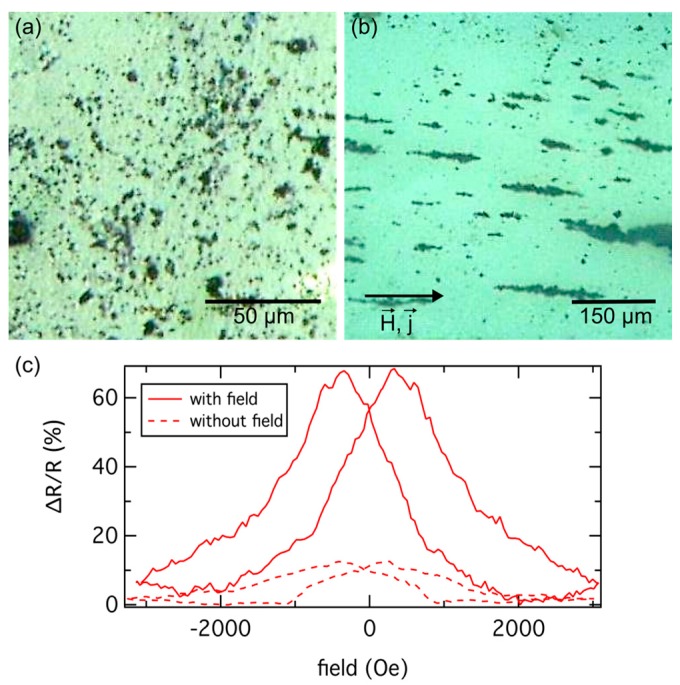
Optical microscope pictures of carbon coated 18 nm sized Co particles in an agarose gel prepared without (**a**) and with a homogenous (**b**) external magnetic field applied during the cooling process. The particles clusters are randomly distributed in the sample without field, while particle chains are formed during the influence of the homogenous field. The granular GMR is measured at room temperature for both samples and shown in (**c**) (data taken from from [123] © IOP Publishing. Reproduced with permission. All rights reserved).

Vargas *et al.* established a model to simulate the dipole coupling between ferromagnetic particles and its influence on the granular GMR [122]. They showed that the particles couple ferromagnetically in the near field, while in the far field an antiferromagnetic coupling is present. Considering a model system of two parallel particle chains with particle moments aligned in one direction within each chain, but opposite to the orientation of the moments of the adjacent chain, a 20% higher GMR has been expected compared to non-interacting nanoparticles. In order to realize such a nanoparticular GMR model system Meyer *et al.* have incorporated carbon coated Co nanoparticles into conductive agarose gels as a non-magnetic matrix [123]. These systems allow an alignment of ferromagnetic Co particles along magnetic field lines of an applied external field. The agarose gel has been heated above the melting point and the nanoparticles are spread in the liquid phase of the gel. During cooling of the gel below the gelling temperature an external magnetic field can be applied. Thus, this technique allows to trigger different particle arrangements in the conductive matrix, which are fixed after the gel’s solidification. Thereby, a variation of the GMR characteristic with every measurement caused by a change of particle positions during switching the external field, which can be observed in the case of a liquid gel matrix like a glycerin-water mixture, can be prevented [123]. Optical microscope images of a sample prepared without and with the influence of a homogenous magnetic field during the cooling process are shown in Figure 17a,b, respectively. A comparison of the corresponding GMR measurements performed at room temperature is given in Figure 17c. The impact of particle arrangement on the nanoparticular GMR effect can be seen clearly. The higher GMR amplitude in the case of the field cooled sample, compared to the sample with the randomly distributed particles, can be attributed to the larger particle volume fraction along the current path, when the current is applied parallel to the field [123].

**Figure 18:**
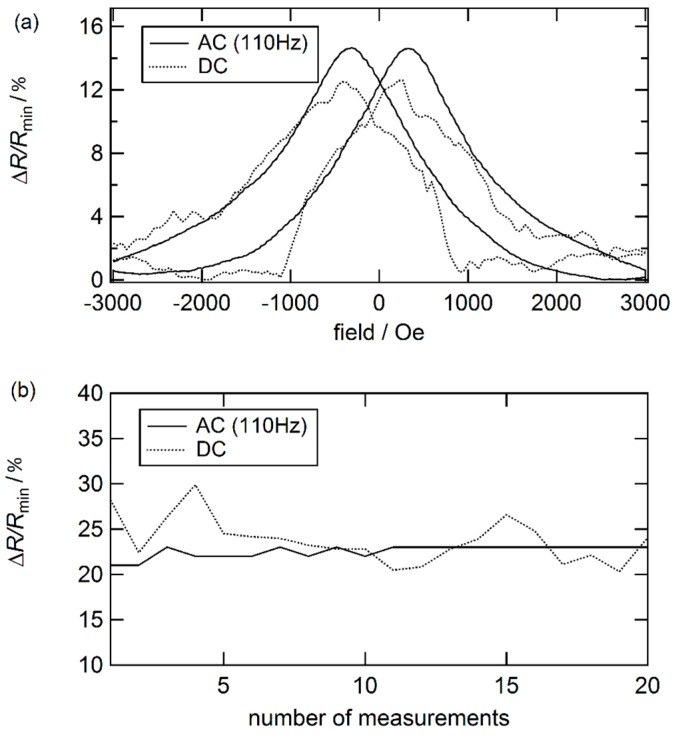
(**a**) Comparison of a nanoparticular GMR measurements at room temperature with DC and AC at 110 Hz; (**b**) The GMR effect development over a number of measurements for DC and AC at 110 Hz (data taken from [124]).

However, the particle density inside these particle superstructures is different. Hence, the dipole coupling inside these superstructures varies as shown by spin-dynamic simulations for a homogenous and a rotating field sample [27]. As a higher particle density is present in the rotating field sample, the interparticle distance is smaller and therefore, more and larger areas of ferromagnetic coupled particles are present compared to the homogenous field sample. As suggested by Vargas *et al.*, these different dipole couplings may have an additional influence on the granular GMR effect as well [122]. To further improve the stability of the agarose gel based nanoparticular GMR characteristics, it is recommended to use an alternating current (AC) instead of a direct current (DC) (compare Figure 18). In doing so, the electrolysis of the ions in the gel and the buildup of electrical double layers are inhibited [123]. This results in an enhancement of reproducibility of nanoparticular GMR effects and therefore, opens a way to realize printable, high sensitivity sensors without the need of photo- or e-beam lithography [67].

## 4. Conclusions

We have shown that the GMR effect occurs in magnetic materials ranging from heterogeneous bulk systems over multilayered thin films to magnetic nanoparticles, synthesized by bottom-up methods. The microstructural as well as magnetic features have found to be crucial to trigger full potential of the GMR effect in all systems. For the future-oriented nanoparticular GMR systems, we have shown that an extensive control of the particle arrangement and magnetic configuration will be the key to a successful establishment of printable detection devices in industrial applications.

## Abbreviations

The following abbreviations are used in this manuscript:

GMR	Giant magnetoresistance
CIP	Current in plane
CPP	Current perpendicular to plane
RKKY	Ruderman-Kittel-Kasuya-Yoshida (interaction)
AMR	Anisotropic magnetoresistance
DC	Direct current
AC	Alternating current
TEM	transmission electron microscopy
AFC	Antiferromagnetic coupling
APT	Atom probe tomography
